# The Nutritional Quality of Broiler Meat Through Dietary Inclusion of Food-Waste–Derived Full-Fat Black Soldier Fly Larvae

**DOI:** 10.3390/foods15111966

**Published:** 2026-06-02

**Authors:** Mohammad S. Alafif, Louwrens C. Hoffman, Faris F. Almutiri, Daniel Cozzolino, Eugeni Roura, M. Reza Abdollahi, Elham A. Soumeh

**Affiliations:** 1 School of Agriculture and Food Sustainability, The University of Queensland, Gatton Campus, Gatton, QLD 4343, Australia; msalafif@uj.edu.sa; 2 Department of Biological Sciences, Faculty of Sciences, University of Jeddah, Jeddah 21959, Saudi Arabia; ffnalmetere@uj.edu.sa; 3 Queensland Alliance for Agriculture and Food Innovation, The University of Queensland, St Lucia Campus, Brisbane, QLD 4072, Australia; louwrens.hoffman@uq.edu.au (L.C.H.); d.cozzolino@uq.edu.au (D.C.); e.roura@uq.edu.au (E.R.); 4 Department of Animal Sciences, Faculty of AgriSciences, Stellenbosch University, Stellenbosch 7602, South Africa; 5 Monogastric Research Centre, School of Agriculture and Environment, Massey University, Palmerston North 4442, New Zealand; m.abdollahi@massey.ac.nz; 6 A2Z Poultry Feed Dynamikz, 69100 Lyon, France

**Keywords:** Chickens, fatty acids, insects, meat quality, omega-3 polyunsaturated fatty acids

## Abstract

This study evaluated breast meat quality of broiler chickens following dietary inclusion of full-fat Black soldier fly (*Hermetia illucens*) larvae (BSFL) sourced from three food-waste production sites in a nutritionally balanced diet. Broilers were fed diets containing 0%, 3%, 6%, or 9% BSFL sourced from 3 different facilities in a 3 × 4 factorial design. At 42 days of age, breast meat samples were collected for evaluation of physicochemical traits, chemical composition, amino acid, and fatty acid profiles. Inclusion of dietary BSFL had no adverse effects on key meat quality parameters, including water-holding capacity, pH, color, cooking loss, or shear force. Breast meat protein content increased significantly in broilers fed the 9% BSFL diet compared with the control, while essential amino acid composition remained unchanged across treatments. In contrast, BSFL inclusion substantially modified the fat profile of breast meat, characterized by enrichment of short- and medium-chain saturated fatty acids, increased eicosapentaenoic acid, reduced ω-6 polyunsaturated fatty acids, and an improved ω-3/ω-6 ratio. These results demonstrate that food-waste-derived full-fat BSFL can be incorporated into broiler diets at levels up to 9% without compromising breast meat quality, while enhancing its nutritional fat profile and protein content.

## 1. Introduction

Chicken meat is among the most widely consumed meats globally, with per capita consumption continuing to rise due to its affordability, versatility, and perceived leanness and healthfulness as an animal protein source [1]. Compared with red meats such as beef or pork, chicken contains lower levels of total fat and saturated fatty acids (SFA), making it popular among consumers seeking healthier dietary patterns [2]. As demand increases, attention has turned toward production strategies that not only maintain performance but also enhance the nutritional value of poultry meat.

The nutritional quality of poultry meat is influenced by the dietary fat profile. Modifying the fatty acid composition of broiler diets can alter intramuscular levels of SFA, monounsaturated fatty acids (MUFA), and polyunsaturated fatty acids (PUFA), with direct implications for human health outcomes related to inflammation, cardiovascular disease, and metabolic function [3,4]. Improving the ω-3/ω-6 PUFA ratio in poultry meat is particularly desirable, as Western diets typically contain excessive ω-6 fatty acids relative to ω-3 fatty acids, a pattern associated with chronic low-grade inflammation and increased cardiometabolic risk.

Black soldier fly (*Hermetia illucens*) larvae (BSFL) have emerged as a promising ingredient for poultry diets due to their favorable nutritional profile and sustainability benefits. Full-fat BSFL typically contain 36.2–65.5% crude protein and 30–40% fat (dry matter basis) [5,6]. The BSFL also provides essential amino acids, key minerals such as calcium and phosphorus, and beneficial fatty acids, including lauric acid, which has antimicrobial properties [6,7]. As BSFL efficiently converts food waste into nutrient-rich biomass, they simultaneously reduce landfill burden and provide a protein source that facilitates the integration of animal production into the circular economy concept [8].

Despite promising nutritional benefits, few studies have assessed the effects of dietary inclusion of BSFL, particularly in full-fat form, on chicken meat quality. Previous studies have shown that the inclusion of BSFL can modify the fatty acid composition of chicken meat. Poultry meat quality is influenced by diet composition [9,10]. For example, feeding 8% of BSFL enhanced meat fatty acid profiles by increasing mono- and polyunsaturated fats, but decreased omega-3 levels in chicken meat [11], while replacing up to 15% of soybean meal with defatted BSFL decreased meat moisture, increased protein, and altered fatty acid composition without negatively affecting carcass or meat quality [12,13]. However, the magnitude and direction of these effects depend heavily on the nutrient profile of the larvae’s growth substrate. Because BSFL composition varies according to the rearing substrate [14], variability in protein, fat, and fatty acid composition among BSFL batches originating from its growth media, may lead to inconsistent nutritional outcomes in poultry meat [8]. This variability represents a major challenge for the large-scale commercial adoption of food-waste–derived BSFL in poultry production, where consistency in feed quality and final meat characteristics is essential. Therefore, evaluating larvae produced from different food-waste is essential for understanding their potential to enhance the nutritional value of chicken meat.

To date, no study has compared multiple BSFL production sites using food-waste inputs to assess their differential effects on the nutritional quality of broiler meat, including amino acids, fatty acids, and health-related indices. Addressing this gap is critical for optimizing insect-based feed ingredients not only for sustainability but also for delivering improved nutritional outcomes relevant to human health.

The present study investigated the impact of full-fat BSFL produced at three production sites, in which food waste was used as the rearing substrate, on the nutritional composition of broiler breast meat, focusing on fatty acids, amino acids, and consumer-relevant meat-quality indicators. By linking substrate-driven variation in BSFL biomass to meat nutritional outcomes, this work provides new insights into the potential of food-waste–derived BSFL to enhance the value of chicken meat while supporting more sustainable feed production systems.

## 2. Materials and Methods

The animal experiments and procedures conducted in this research project received approval from the Animal Ethics Committee of the University of Queensland (Approval No: 2023/AE000309).

### 2.1. Black Soldier Fly Larvae Preparation

The BSFL were supplied by a commercial food waste processing facility (Goterra Pty Ltd., Canberra, Australia) and sourced from three commercial production facilities (BSFL-A, BSFL-B and BSFL-C). Facility A used supermarket waste, mainly bread, meat, salads, pastries, chips, and dairy products, while Facility B used waste from childcare centers, including salads, bread, and a variety of cooked meals. Facility C used a mixture of supermarket and coffee waste collected from local coffee shops. All food waste was de-packaged and homogenized using an industrial homogenizer prior to feeding the larvae. Larvae were fed *ad libitum* and harvested at the 5th–6th instar stage. Feed moisture was maintained above 70%, monitored with an A&D Weighing MF50 moisture analyzer (A&D Company, Limited, Tokyo, Japan). Rearing conditions were maintained above 25 °C, with ambient humidity monitored using temperature and moisture sensors, in accordance with standard facility protocols. Post-harvest, larvae were blanched at 70 °C for 1 min, microwave-dried at 75 °C for 20 min, and ground to pass through a 6.0 mm screen.

### 2.2. Experimental Animals, Husbandry and Diets

A total of 576 one-day-old Ross 308 male broiler chickens were obtained from a commercial hatchery (Aviagen Australia Pty Ltd., Goulburn, NSW, Australia) and transferred to the Queensland Animal Science Precinct (QASP, Gatton Campus, The University of Queensland). Sample size was determined using a priori power analysis conducted in Minitab (version 21, Minitab LLC, State College, PA, USA) based on a one-way ANOVA design with α = 0.05 and statistical power of 0.80. Body weight gain was used as the primary response variable for the power analysis. The analysis assumed a standard deviation of 300 and a maximum detectable difference of 198.977 between treatment means, corresponding to an approximate standardized effect size of 0.66. Upon arrival, birds were individually weighed and randomly allocated to 72 floor pens (1 m × 1 m) in a 3 × 4 factorial arrangement with three BSFL sources and four dietary inclusion levels (0%, 3%, 6% and 9%) generating twelve iso-caloric, and iso-amino acidic experimental diets. Each dietary treatment was replicated six times, with eight birds per pen (*n* = 48 per treatment). The treatments and replicates were randomly allocated to the pens. A common starter diet was fed from day 1 to 10; thereafter, the experimental diets were fed during the grower (days 11–28) and finisher (days 29–42) phases, for a total of 32 days. The amino acid profiles of the full-fat BSFL and experimental diets are presented in Table 1 and Table 2, and fatty acid profiles in Table 3 and Table 4, respectively. Birds were monitored daily and humanely euthanized if any signs of sickness were detected. They had *ad libitum* access to feed and water and were reared under controlled environmental conditions in accordance with breeder guidelines until slaughter at 42 days of age.

On day 42, all birds were weighed, and one bird per pen (6 birds per treatment) with a body weight close to the pen average was selected for slaughter at the QASP facility. Live weight was recorded prior to slaughter. Birds were electrically stunned, exsanguinated, scalded at 60 °C, de-feathered, and eviscerated. After processing, carcasses were refrigerated at 4 °C for approximately 18 h, after which cold carcass weight was recorded. Dressing percentage was calculated as:

$$\mathrm{Dressing}\;\mathrm{percentage}=\left(\frac{cold\;carcass\;weight}{Live\;weight\;}\right)\times 100$$

Following this refrigeration period, breast meat samples were collected for analysis.

### 2.3. Meat Quality Analysis

#### 2.3.1. Water Holding Capacity (WHC)

The breast meat samples, weighing 1.000 g on an analytical scale with 0.005 g accuracy, were cut and placed between two labelled filter papers (Lasec, Cape Town, South Africa; grade 292, diameter 90 mm, part number FLAS3205090). The meat was pressed between Perspex plates (standard pressure: 588 N) for 1 min. After allowing the filter paper to dry for approximately 10 min to enhance image clarity, an image of the filter paper was taken, showing the drip area and the pressed meat area. A ruler was placed next to each sample in the image, for scale reference. The drip area (indicating % loose water) was measured using ImageJ software (version 0.6.0; https://imagej.net/ij), and this procedure was repeated for all samples to calculate the WHC as [15]:

Water Holding Capacity=Total Water−Loose Water

$$\%Loose\;Water=\left(b-a\right)\times \frac{0.0084}{1}\times 100\%$$

b: area enclosed by the outer front (cm^2^)

a: area enclosed by the inner front (cm^2^)

#### 2.3.2. Breast Meat pH

A portable pH meter (Hanna HI 98163, Keysborough, VIC, Australia) was calibrated with pH buffers at 4 and 7 according to the manufacturer’s instructions, with recalibration performed after every six readings. For each breast sample, the pH probe, which included a temperature-compensating point, was inserted 1 cm into the center of the breast tissue, and the readings were recorded. The pH probe was cleaned between samples, following the manufacturer’s guidelines.

#### 2.3.3. Meat Colour

To assess meat color, the left half of each chicken’s breast was utilized and the color attributes measured after a 20 min bloom period with a Chromameter CR-400/410 (Thermo Fisher Scientific Pty Ltd., Waltham, MA, USA), which was configured for diffuse illumination at a 0° viewing angle, including the specular component, and then set to the CIE standard observer (2°). The chromameter was calibrated on a white tile according to the manufacturer’s guidelines. Measurements were taken at optical infinity on the chicken breast meat, with color readings recorded at three distinct locations on the cut, perpendicular to the muscle fibers, surface. Values for L* (Lightness), a* (Redness), and b* (Yellowness) were recorded. To obtain Hue and chroma for a more precise determination of meat color, the following equations were used:

$$Chroma\;(C^{*})=\sqrt{{\left(a^{*}\right)}^{2}+({b^{*})}^{2}}$$

$$Hue\;angle\;\left(h^{ab}\right)=\;tan-1\left(\frac{b^{*}}{a*}\right)$$

#### 2.3.4. Cooking Loss (CL)

A slice of chicken breast meat with an approximate weight of 70 g, was cut from the breast center, weighed and then placed in a sealed thin-walled plastic bag. The bag was submerged in an 80 °C water bath for 40 min to ensure thorough cooking. After cooking, the samples were chilled, the water drained from the bag, and the sample gently blotted dry with absorbent paper before weighing again. The CL was calculated as follows [15]:

$$\%\;Cooking\;Loss=\frac{\left(weight\;before-weight\;after\right)}{\left(weight\;before\right)}\times 100$$

#### 2.3.5. Shear Force (SF)

Cooked breast meat samples that were used for CL, were cooled in the refrigerator until 4 °C. To evaluate meat tenderness, samples of cooked meat, each with a surface area of 1 cm^2^, were cut parallel to the meat’s longitudinal axis. The SF values were then measured using an Instron 5543 model (15 Stud Road, Baywater, Melbourne, VIC, Australia) equipped with a Warner–Bratzler blade (1 mm thick with a triangular opening of 13 mm at the widest point, 15 mm high, and 60 mm long, with a 60° cutting angle). The maximum SF values (N) were recorded for each sample at a crosshead speed of 33.3 mm/s. Three cuts were made from each sample, and the average value was used for statistical analyses.

#### 2.3.6. Chemical Composition Analysis

The dry matter content was determined by drying the samples in an oven at 100 °C for 24 h, in accordance with Association of Official Analytical Chemists International (AOAC) Method 930.16 [16]. Ash content was measured by combusting moisture-free meat samples in a muffle furnace at 500 °C for 6 h, according to AOAC Method 942.05 [17]. Fat was extracted using a chloroform/methanol (2:1, *v*/*v*) solvent system [18]. The defatted samples were subsequently dried at 60 °C to a constant weight (approximately 4 h). Fat content was calculated as the difference between the dry mass before and after extraction. Protein content was determined by measuring nitrogen concentration in the defatted, dried samples using a LECO CN928 carbon/nitrogen combustion analyzer at 1100 °C, with approximately 0.3 g of sample. Crude protein was calculated as nitrogen × 6.25. Neutral detergent fibre and acid detergent fibre contents in BSFL samples were determined using an Ankom200 Fibre Analyser (ANKOM Technology, Macedon, NY, USA) according to [19]. The chitin content was estimated according to the equation [20]:

Chitin=Aciddetergent fibre−Acid detergent lignin

#### 2.3.7. Amino Acid and Fatty Acid Analysis

The amino acid composition of diet and breast meat samples were determined following acid hydrolysis and chromatographic separation. The freeze-dried samples were hydrolyzed in 6 M hydrochloric acid at 110 °C for 24 h under nitrogen to prevent oxidative degradation, in accordance with AOAC guidelines (Methods 982.30 and 994.12) [21]. Following hydrolysis, samples were neutralized and derivatized prior to chromatographic analysis. Amino acids were separated using high-performance liquid chromatography (HPLC) with UV/fluorescence detection and quantified against authenticated external amino acid standards. Analyses were performed using a Shimadzu Nexera X2 UHPLC system (Shimadzu Corporation, Kyoto, Japan) coupled to a Shimadzu 8030 mass spectrometer, applying a modified Waters AccQ-Tag derivatization method (Waters Corporation, Milford, MA, USA). Sulfur-containing amino acids (methionine and cysteine) were quantified (mg/g DM) following performic acid oxidation, in accordance with AOAC Method 994.12 [22].

The fatty acid profiles of the experimental diets and breast meat samples were determined after fat extraction from freeze-dried material using a chloroform:methanol (2:1, *v*/*v*) solvent system under low-light conditions to prevent carotenoid degradation, following a modified Folch method [23]. Fatty acids were subsequently converted to fatty acid methyl esters (FAME) using standard base- and acid-catalyzed methylation procedures, in accordance with AOAC Method 996.06 [24] and ISO 12966-2:2017 [25]. FAME were analyzed by gas chromatography equipped with a flame ionization detector (HS-GC/FID, Agilent, Les Ullis, France) and a polar capillary column. Individual fatty acids were identified by comparison with authenticated FAME standards and quantified (mg/100 g DM) using calibrated response factors.

### 2.4. Statistical Analysis

A two-way analysis of variance (ANOVA) was performed using the General Linear Models (GLM) procedure of SAS (2016) to evaluate the effects of BSFL production site, dietary inclusion level, and their interaction on the measured parameters. BSFL production site and inclusion level were included as fixed effects. As dietary treatments were applied at the pen level, pen was considered the experimental unit. For meat quality and compositional analyses, one bird was sampled from each pen; therefore, each sampled bird represented an independent replicate pen, avoiding pseudo-replication.

Least squares means were calculated for BSFL production site, inclusion level, and their interaction, and mean separations were performed using Tukey’s adjustment for multiple comparisons. Statistical significance was declared at *p* < 0.05.

Model assumptions and diagnostics were assessed using predicted values, raw residuals, studentized residuals, and Cook’s distance generated from the GLM procedure to identify potential outliers and influential observations. No data points were excluded unless they met the predefined statistical diagnostic criteria.

## 3. Results

### 3.1. Meat Quality Traits

The effects of full-fat BSFL on broiler chickens’ breast meat quality parameters and proximate analysis are presented in Table 5 and Table 6, respectively.

No significant interactions between BSFL production site and inclusion rate were observed for any meat quality parameters, including LW, cold carcass traits, dressing percentage, pH, WHC, SF, CL, or color attributes (L, a, b*, hue angle, chroma). For the main effect of BSFL production sites, most traits did not differ among the three sources (BSFL-A, BSFL-B, and BSFL-C). However, redness (a*) tended to be higher in BSFL-C (3.13) compared with BSFL-A (2.56) and BSFL-B (2.69). Increasing inclusion levels of full-fat BSFL had no significant effect on any meat quality trait.

No interactions between BSFL production site and inclusion rate were detected for the chemical composition of the broilers’ breast meat. However, the chemical composition was influenced by BSFL inclusion rate. Protein content increased significantly from 20.0% in the control group (0%BSFL) to 21.7% at the highest inclusion level (9%BSFL). In contrast, moisture, fat, and ash contents were unaffected by inclusion rate. For the main effect of BSFL production site, protein and fat contents did not differ, although ash tended to be higher in BSFL-C (1.34%) compared with BSFL-A (1.22%) and BSFL-B (1.23%).

### 3.2. Amino Acid Profiles

The effects of the BSFL production site and inclusion level on the essential amino acid and non-essential amino acid composition of broiler breast meat are presented in Table 7 and Table 8, respectively. Dietary inclusion of BSFL had minimal impact on the amino acid composition of broiler breast meat. No essential amino acid was significantly affected by the BSFL production site or the inclusion rate.

For non-essential amino acid (Table 8), several production site-related differences were observed. Glycine concentrations differed significantly, with higher levels in birds fed BSFL-C (31.7 mg/g DM) compared with BSFL-B (30.7 mg/g DM). Similarly, proline varied among production sites, with birds receiving BSFL-A showing the highest concentration (27.0 mg/g DM) and birds receiving BSFL-B showing the lowest (26.1 mg/g DM). Cysteine tended to be higher in BSFL-A and BSFL-C compared with BSFL-B. Additionally, Serine and Tyrosine showed tendencies toward production site effects, with BSFL-C generally having the highest values. Across inclusion levels, glycine was the only significantly affected non-essential amino acid. Glycine concentration was higher at 3% BSFL inclusion (32.1 mg/g DM) compared with 6% (30.8 mg/g DM) and 9% (30.8 mg/g DM).

### 3.3. Fatty Acid Profiles

#### 3.3.1. Saturated Fatty Acids (SFA)

The effects of BSFL production site and inclusion level on the SFA composition of broiler breast meat are presented in Table 9. A significant interaction between BSFL production site and inclusion level was observed for lauric acid (C12:0). Across all production sites, C12:0 increased with increasing inclusion level, ranging from 1.47 (0%), 23.08–42.96 (3%), 45.48–62.08 (6%), and 65.24–93.51 mg/100 g DM (9%). No significant interactions between BSFL production site and inclusion level were detected for the other individual SFA. For the main effect of the BSFL production site, capric acid (C10:0) differed significantly among treatments, with BSFL-A and BSFL-B showing higher values than BSFL-C. No other individual SFA or total SFA (ΣSFA) were affected by the BSFL production site.

In contrast, the inclusion level of BSFL significantly affected several individual saturated fatty acids. C10:0 increased from undetectable levels at 0% inclusion to 2.72 mg/100 g at 9% inclusion. C12:0 increased markedly by approximately 54-fold, while myristic acid (C14:0) increased by approximately 3.6-fold between 0% and 9% inclusion levels. ΣSFA showed a trend toward an increase with inclusion rate, rising from 498.06 mg/100 g at 0% to 628.48 mg/100 g at 9% inclusion.

#### 3.3.2. Monounsaturated Fatty Acids (MUFA)

The effects of BSFL production site and inclusion level on MUFA composition of broiler breast meat are presented in Table 10. There were no significant BSFL production site and inclusion rate interactions detected for individual MUFA or total. Likewise, no significant main effects of BSFL production site were observed for individual MUFA or ΣMUFA. However, a tendency was observed for palmitoleic acid (16:1n-7), with BSFL-B (66.83 mg/100 g) showing higher values than BSFL-A (53.23 mg/100 g) and BSFL-C (53.86 mg/100 g).

Similarly, BSFL inclusion level significantly influenced several MUFA components. Inclusion rate had a significant effect on myristoleic acid (14:1n-5), which increased with increasing BSFL inclusion, rising from 1.67 mg/100 g at 0% to 5.11 mg/100 g at 9% inclusion, representing an approximately 3.1-fold increase. Similarly, palmitoleic acid (16:1n-7) increased significantly with inclusion level, from 43.62 mg/100 g at 0% to 65.92 mg/100 g at 9% inclusion. Other MUFA, including oleic acid (18:1n-9 cis), vaccenic acid (18:1n-7), gondoic acid (20:1n-9), erucic acid (22:1n-9), and nervonic acid (24:1n-9), as well as total MUFA, were not affected by inclusion level.

#### 3.3.3. Polyunsaturated Fatty Acids (PUFA)

The effects of BSFL production site and inclusion level on the PUFA content of broiler breast meat are presented in Table 11. A significant interaction between BSFL production site and inclusion level was observed for the ω-3/ω-6 ratio. Across all BSFL production sites, the ω-3/ω-6 ratio increased progressively with increasing inclusion level, with the highest value observed at 9% inclusion. In particular, the ω-3/ω-6 ratio increased from 0.14 at 0% to 0.18–0.19 at 9% inclusion, corresponding to an approximately 1.3-fold increase, with BSFL-A and BSFL-B showing greater increases than BSFL-C. For the main effect of the BSFL production site, no significant differences were detected for individual PUFA, total PUFA, or total ω-3 and ω-6 PUFA. However, the ω-3/ω-6 ratio differed significantly among BSFL production sites, with BSFL-C showing a lower ratio compared with BSFL-A and BSFL-B.

BSFL inclusion level significantly affected several PUFA components. Inclusion rate reduced α-linolenic acid (ALA; 18:3n-3) from 32.31 mg/100 g DM at 0% to 25.20 mg/100 g DM at 9% inclusion, representing an approximately 22% reduction. Eicosapentaenoic acid (EPA; 20:5n-3) increased with increasing BSFL inclusion level, rising from 2.64 mg/100 g at 0% to 4.81 mg/100 g at 9% inclusion, corresponding to an approximately 1.8-fold increase.

Linoleic acid (18:2n-6) increased at 3% inclusion (405.29 mg/100 g DM) relative to 0% (371.47 mg/100 g DM), but declined at higher BSFL inclusion levels, reaching 279.89 mg/100 g at 9% inclusion (~0.75-fold vs. 0%). Eicosatrienoic acid (20:3n-6) decreased with increasing BSFL inclusion, from 60.90 mg/100 g DM at 0% to 48.73 mg/100 g DM at 9% inclusion (~0.80-fold). Similarly, arachidonic acid (20:4n-6) declined from 0.79 mg/100 g DM at 0% to 0.26 mg/100 g DM at 9% inclusion (~0.33-fold), and 22:4n-6 decreased from 4.05 mg/100 g DM to 2.45 mg/100 g DM.

Total ω-6 PUFA decreased significantly with increasing BSFL inclusion level, from 447.29 mg/100 g at 0% to 340.41 mg/100 g at 9% inclusion (~0.76-fold; 24% reduction). The ω-3/ω-6 ratio increased from 0.14 mg/100 at 0% to 0.17 mg/100 g at 9% inclusion (~1.21-fold). Total PUFA increased at 3% inclusion compared with the control (544.71 vs. 506.21; ~1.08-fold), but decreased at higher inclusion levels, reaching the lowest value at 9% inclusion (397.72; ~0.73-fold relative to 3%).

## 4. Discussion

### 4.1. Meat Quality

Meat quality and physicochemical parameters are critical in poultry production, especially in broilers, as they directly affect consumer acceptance, market value, and processing efficiency. No effects between BSFL production site source and inclusion rate on any of the measured carcass traits and meat quality parameters were noted. Furthermore, for the main effect of BSFL production site, no differences were observed among the three sources for all parameters. Consistent with these results, Schiavone et al. [12] found that the inclusion of up to 10% BSFL did not affect meat quality parameters, including CL. Likewise, no significant changes in meat pH, color, or WHC in broilers fed diets with up to 15% BSFL were reported in an earlier study [13]. Other studies have reported that BSFL does not impair the physicochemical indices of meat, including color, pH, and WHC in broiler chicken [13,26,27]. Saidani et al. [28] reported no significant differences in meat quality parameters, including pH, myoglobin, WHC, CL, and chemical composition (DM, ash, and ether extract).

However, in this study, a tendency for greater redness was observed in the BSFL-C compared to BSFL-A and BSFL-B (a* value = 3.13 Vs 2.56 Vs 2.69, respectively). Previous studies have reported increased breast meat redness in broiler chickens following the dietary inclusion of BSFL or BSFL-derived oils [12,28,29]. Schiavone et al. [12] found that the inclusion of defatted BSFL increased the redness of chicken breast meat. Similarly, Saidani et al. [28] reported an increase in redness in the breast meat of broilers fed BSFL. In contrast, Lee et al. [30] observed a linear decrease in redness and yellowness with increasing BSFL inclusion levels in the diet.

Meat color can be influenced by dietary ingredients, particularly through the accumulation of pigments in intramuscular fat. In the case of BSFL, this effect may vary depending on the form (whole larvae, full-fat or defatted meal, or oil) and origin (rearing substrate) of the larvae used. According to Aprianto et al. [29], the increase in meat redness may be linked to the presence of xanthophyll pigments accumulated in BSFL reared on vegetable- and fruit-based substrates. This could partly explain the higher meat redness observed in the birds fed BSFL-C, where coffee grounds were a major component of the rearing substrate. Because the chemical composition of BSFL is largely influenced by its rearing substrate, this can affect its fat and pigment profiles, ultimately influencing broiler meat color. Although coffee contains pigments such as polyphenols, melanoidins, and chlorogenic acids, these are not fat-soluble like xanthophylls and are therefore less likely to be deposited in muscle tissue and directly influence redness (a*) or yellowness (b*) in the meat. This aspect warrants further research. Moreover, it has been reported that diets richer in carotenoid pigments, such as corn, contributed to the yellow skin coloration in Label Rouge chickens, as the carotenoids accumulate in the intramuscular fat Baéza et al. [31]. Meat color is a key visual indicator of quality and freshness in poultry, particularly chicken, and significantly influences consumer acceptance. In addition to dietary pigments, factors such as pre-slaughter stress, genetics, growth rate, pH, and diet composition can also affect meat color.

Regarding the chemical composition of broiler breast meat, the source of BSFL did not significantly affect protein or fat content. However, there was a tendency toward higher ash levels in the BSFL-C (1.34%) than in BSFL-A (1.22%) and BSFL-B (1.23%). One possible explanation for the elevated ash content in the BSFL-C is that the larvae were reared on food waste rich in spent coffee grounds, which contributed to greater mineral retention in the larvae and, subsequently, in the broiler meat. Previous studies have indicated that the composition and processing of BSFL can influence the meat ash content. Murawska et al. [32] observed a decrease in ash content in broiler meat with increasing levels of full-fat BSFL. On the other hand, Cullere et al. [33] reported that defatted BSFL meals have higher mineral content, which may result in higher ash levels in animal tissues. Popova et al. [34] reported no significant changes in meat ash content regardless of whether full-fat or partially defatted larvae was fed to chickens. These results indicate that both the substrate on which BSFL are reared and their processing method (e.g., defatting) can influence their nutritional contribution to poultry meat, particularly with respect to mineral deposition.

The protein content of broiler breast meat observed in this study aligns with previously reported ranges by Altmann et al. [26] (20.52–21.07%), and Murawska et al. [32] (22.19–22.97%). However, in the present study, dietary inclusion rate of BSFL had a significant effect on the breast meat protein content, with a notable increase observed between the 0% and 9% inclusion levels. The highest protein value was recorded at 9% inclusion rate (21.7%), significantly higher than the control group with lowest protein content (20.0%). These findings are consistent with Schiavone et al. [12], which reported a linear increase in protein content with increasing BSFL inclusion, whereas fat and ash contents remained largely unaffected. Likewise, Murawska et al. [32] found a quadratic increase in meat protein content in broilers fed full-fat BSFL, and Aprianto et al. [29] observed a significant improvement in meat protein with dietary supplementation of BSFL oil calcium salt.

Protein content plays a key role in meat WHC, as higher meat protein levels are typically associated with improved water retention and reduced CL [29]. However, in this study, although the 9% inclusion rate had a higher crude protein (21.7%) than the 0% inclusion rate (20.0%), its WHC was similar (72.6% vs. 73.2%), and CL was slightly higher (26.8% vs. 25.8%). This inverse pattern suggests that other factors, such as muscle structure or fat distribution, may influence WHC and CL. As noted by Hutabarat et al. [35], while WHC and CL are closely related, the relationship is not always strictly linear. On the other hand, Cullere et al. [33] found no significant differences in moisture, protein, fat, or ash content of broiler meat fed with BSFL larvae. Similarly, Saidani et al. [28] observed no significant changes in the chemical composition of broiler meat fed diets containing 5% full-fat BSFL compared with controls. Moreover, Somparn, et al. [36] found that the use of BSFL oil as a feed ingredient for broiler chickens did not significantly affect the meat color (L*, a*, and b*), drip loss, CL, SF, and hardness. These variations highlight the influence of factors such as inclusion level, diet formulation, and the physical form (e.g., full-fat vs. defatted) of the BSFL on meat quality outcomes.

### 4.2. Meat Amino Acids Profile

The present study demonstrates that dietary inclusion of food-waste–derived BSFL has minimal impact on the amino acid composition of broiler breast meat, particularly for essential amino acids. Neither the BSFL production site nor the inclusion level affected the concentration of any essential amino acid, indicating that BSFL can be incorporated into broiler diets without compromising meat protein quality. These findings are broadly consistent with previous reports showing limited effects of BSFL inclusion on the essential amino acid profile of poultry meat. Cullere et al. [37] reported that inclusion of 10% and 15% defatted BSFL in Japanese quail diets did not alter most essential amino acid concentrations, except for an increase in threonine at the 15% inclusion level. Similarly, Srikha et al. [38] observed increases in threonine at 10% BSFL inclusion and lysine at 10–12% inclusion in Thai native chickens. In contrast, de Souza Vilela et al. [39] reported a reduction in lysine concentration in broiler breast meat when BSFL was included in the diet.

In contrast to essential amino acids, several non-essential amino acids showed significant or near-significant differences among BSFL production sites. Glycine concentration was significantly higher in broilers fed BSFL-C compared with BSFL-B, while proline was highest in birds receiving BSFL-A. Additionally, cysteine, serine, and tyrosine showed tendencies toward production site-related effects, with BSFL-C generally associated with numerically higher values. Unlike protein content, which is largely influenced by the chemical composition of the rearing substrate, the amino acid composition of body tissues does not directly mirror dietary amino acid profiles [37]. This is because tissue amino acid deposition is tightly regulated by multiple post-ingestive processes, including digestion and absorption efficiency, intestinal transport, metabolic partitioning, and genetic and physiological regulation of protein synthesis and turnover [40,41]. Consequently, changes in dietary amino acid supply do not necessarily result in proportional changes in meat amino acid composition.

From a human nutrition perspective, the observed variation in selected non-essential amino acids may be relevant despite their classification as non-essential. Glycine and proline are major components of collagen and connective tissue and contribute to muscle structure [42,43]. Glycine also contributes to anti-inflammatory pathways and metabolic regulation, whereas cysteine is a precursor of glutathione synthesis, a key intracellular antioxidant [42,44].

Across inclusion levels, glycine was the only non-essential amino acid significantly affected, with concentrations peaking at 3% inclusion and declining at 6% and 9%. Similar non-linear patterns have been reported previously [39]. Although the exact metabolic pathways responsible for the accumulation of these amino acids in breast muscle are not fully understood, the observed variations likely reflect differences in amino acid metabolism, utilization, and muscle protein turnover rather than direct dietary transfer.

Despite the growing interest in BSFL as a sustainable feed ingredient, studies evaluating the effects of BSFL inclusion in broiler diets on the amino acid composition of broiler meat remain limited. Current evidence suggests that dietary BSFL has minimal influence on meat amino acid profiles; however, these findings provide a valuable foundation for future investigations. Further research is needed to elucidate the effects of BSFL inclusion level and source on the amino acid composition and nutritional quality of broiler meat.

### 4.3. Meat Fatty Acids Profile

The fatty acid composition of foods of animal origin is a key determinant of their nutritional quality and potential health implications for consumers. In the present study, dietary inclusion of food-waste–derived BSFL modified the fatty acid profile of broiler breast meat primarily with respect to inclusion level, whereas the BSFL production site contributed modest but detectable differences in selected fatty acid parameters.

#### 4.3.1. Saturated Fatty Acids

Increasing BSFL inclusion led to dose-dependent increases in medium-chain SFAs, particularly capric (C10:0) and lauric acids (C12:0), while the major long-chain SFAs, palmitic (C16:0) and stearic acid (C18:0), remained unchanged, except for an increase in myristic acid (C14:0). Across all treatments, palmitic acid (C16:0) was the predominant saturated fatty acid, followed by stearic acid (C18:0), which is consistent with the findings of Kierończyk et al. [27] and de Souza Vilela et al. [39]. Notably, the concentrations of these dominant long-chain SFAs were unaffected by the inclusion of BSFL. The absence of changes in these dominant SFAs suggests that the inclusion of BSFL does not adversely affect the primary SFA fraction in broiler meat. In contrast, increasing BSFL inclusion resulted in pronounced, dose-dependent enrichment of capric (C10:0), lauric (C12:0), and myristic (C14:0) acids. This is consistent with previous studies [39,45]. Lauric acid displayed the largest response, increasing approximately 56-fold from 0% to 9% inclusion, while myristic acid increased by approximately 3.8-fold. These changes reflect the intrinsic fat composition of BSFL, which is characteristically rich in medium-chain fatty acids, particularly lauric acid [46]. In line with these individual fatty acid responses, ΣSFA showed a trend toward an increase with increasing BSFL inclusion, rising from 498.06 mg/100 g at 0% to 628.48 mg/100 g at 9% inclusion (≈1.3-fold). By comparison, previous studies have reported a significant increase in ΣSFA in broiler meat following the inclusion of BSFL [39,45]. The increase in ΣSFA was mainly attributable to medium-chain fatty acids, as major long-chain SFAs were unaffected by dietary treatment.

The pronounced increase in medium-chain fatty acids, particularly lauric acid, observed in breast meat further suggests that these fatty acids were efficiently transferred from the insect-based diet into broiler muscle tissue. Medium-chain fatty acids such as lauric acid are metabolized differently from long-chain fatty acids, as they are more readily digested and absorbed and can be transported directly to the liver via the portal circulation for rapid metabolic utilization [47]. In addition, MCFAs are absorbed efficiently by intestinal enterocytes via simple diffusion and are less dependent on bile-mediated emulsification and micelle formation than long-chain fatty acids, thereby contributing to their rapid intestinal uptake and systemic availability [48,49,50]. It was reported that dietary inclusion of BSFL increases circulating concentrations of lauric and myristic acids, thereby supporting the efficient absorption and metabolic availability of these fatty acids following dietary intake [51]. Lauric acid has also been reported to exhibit a lower tendency for adipose accumulation due to its rapid mitochondrial β-oxidation [47,52,53]. Despite this preferential oxidation for energy production, the marked enrichment of lauric acid observed in the present study indicates that a considerable proportion was ultimately incorporated into muscle tissue, suggesting relatively efficient transfer of dietary lauric acid from BSFL into broiler breast meat [39,47]. However, the observed variations in muscle fatty acid composition were unlikely to represent a completely direct linear reflection of dietary fatty acid composition. Previous research has shown that although BSFL-based diets are rich in saturated fatty acids, tissue fatty acid deposition patterns may differ substantially, with selective increases observed for specific fatty acids such as lauric and myristic acids, whereas dominant long-chain saturated fatty acids may remain comparatively stable [39,54]. Similarly, in the present study, palmitic and stearic acids remained relatively stable despite substantial increases in dietary medium-chain fatty acids.

From a human nutrition perspective, it is critical to distinguish between long-chain SFAs, which are strongly associated with elevated low-density lipoprotein (LDL) cholesterol and increased cardiovascular disease risk [55], and short-chain SFAs, which exhibit distinct metabolic properties. Palmitic acid, the most abundant SFA in poultry meat, has consistently been identified as a principal driver of LDL cholesterol elevation [55]. The stability of palmitic and stearic acid concentrations across treatments, therefore, suggests that BSFL inclusion does not exacerbate the SFA fraction most strongly implicated in adverse cardiometabolic outcomes. Medium-chain SFAs, including capric and lauric acids, are metabolized differently from long-chain SFAs. These fatty acids are absorbed directly into the portal circulation and rapidly oxidized in the liver, resulting in their lower propensity for incorporation into adipose tissue [47]. Lauric acid has also been shown to raise high-density lipoprotein (HDL) cholesterol to a greater extent than other SFAs, which may partially offset its LDL-raising effect when compared with total cholesterol [56]. In addition, lauric acid exhibits antimicrobial and immunomodulatory properties, attributes that have generated interest in its potential functional role in human diets [47]. Myristic acid occupies a transitional position between medium- and long-chain SFAs and has been reported to exert a relatively strong effect on LDL-cholesterol levels per unit intake [55,56]. Although myristic acid increased with BSFL inclusion in the present study, its absolute contribution to total saturated fat intake remained considerably smaller than that of palmitic acid. Importantly, the increase in myristic acid occurred without a concurrent increase in palmitic acid, thereby limiting its overall impact on the meat’s saturated fat quality. Although lauric acid content in meat increased substantially in the present study, concentrations of the dominant long-chain saturated fatty acids remain unchanged, which is important within the context of current nutritional recommendations emphasizing limitation of saturated fatty acid intake and improvement of overall dietary fat quality to support cardiovascular health [57,58]. Furthermore, dietary guidelines generally recommend replacing excessive saturated fat intake with unsaturated fatty acids to support cardiometabolic health [58].

The enrichment of ΣSFA observed with higher BSFL inclusion should be interpreted as a qualitative modification rather than a uniform elevation of long-chain saturated fats. The shift toward greater representation of short- and medium-chain SFAs, combined with the stability of palmitic and stearic acids, suggests that BSFL inclusion modifies the SFA profile of broiler breast meat in a manner unlikely to disproportionately increase cardiometabolic risk. When considered within the context of a balanced diet, these changes may be nutritionally neutral or potentially favorable, while simultaneously supporting the development of more sustainable poultry production systems.

#### 4.3.2. Monounsaturated Fatty Acids 

The modulation of MUFA observed in the present study is broadly consistent with previous work investigating the inclusion of full-fat BSFL in boiler diets [39]. Across treatments, oleic acid (18:1n-9 cis) was the dominant MUFA, accounting for the largest proportion of total MUFA. Oleic acid concentration was not affected by BSFL inclusion or BSFL production site, indicating preservation of the principal MUFA fraction that underpins many of the cardioprotective properties associated with poultry meat consumption. In contrast, the inclusion of BSFL significantly increased several MUFAs. Myristoleic acid (14:1n-5) and palmitoleic acid (16:1n-7) increased with increasing inclusion levels (3.2-fold and 1.6-fold increases, respectively), which is consistent with the findings of de Souza Vilela et al. [39] who also reported elevated levels of these fatty acids in the breast meat of broilers fed diets containing 20% BSFL. Palmitoleic acid has been described as a “lipokine” with potential regulatory effects on fat metabolism, insulin sensitivity, and inflammation, and its enrichment may have favorable metabolic implications for consumers [59,60]. Despite changes in individual MUFAs, total MUFA content was unaffected, consistent with other reports [39,45]. From a human nutrition perspective, these findings are broadly favorable and comparable with prior BSFL studies. The preservation of oleic acid, combined with enrichment of palmitoleic acid and other minor MUFAs, reflects that BSFL-fed broiler meat maintains a MUFA profile supportive of cardiometabolic health.

#### 4.3.3. Polyunsaturated Fatty Acids 

The PUFA are key contributors to the nutritional quality of poultry meat due to their established roles in cardiovascular health, immune regulation, and modulation of inflammatory pathways in humans [3,61]. In the present study, dietary inclusion of food-waste–derived BSFL significantly enhanced the PUFA profile of broiler breast meat.

The most nutritionally relevant response to increasing BSFL inclusion was observed in the balance between ω-3 and ω-6 PUFA rather than in total PUFA content per se. Across all BSFL production sites, the ω-3/ω-6 ratio increased progressively with inclusion level, rising from 0.14 in the control diet to approximately 0.18–0.19 at 9% inclusion, representing a ~1.3-fold increase. This improvement in the ω-3/ω-6 ratio was primarily driven by a marked increase in the long-chain ω-3 PUFA eicosapentaenoic acid (EPA; 20:5n-3), which increased from 2.64 mg/100 at 0% inclusion to 4.81 mg/100 at 9% inclusion. This was accompanied by decreases in several ω-6 PUFA with increasing BSFL inclusion, including eicosatrienoic acid (20:3n-6) and arachidonic acid (20:4n-6), contributing to a significant reduction in total ω-6 PUFA (−24% at 9% inclusion).

The EPA is widely recognized for its anti-inflammatory and cardioprotective properties, and higher dietary intake has been associated with reduced cardiovascular disease risk [3,61]. Although the absolute concentrations of ω-3 PUFA in broiler meat remain modest compared with marine sources, poultry meat is consumed frequently and in substantial quantities worldwide. Consequently, even moderate increases in EPA content and improvements in the ω-3/ω-6 ratio may contribute meaningfully to habitual ω-3 intake at the population level. Moreover, enrichment of animal-derived foods with ω-3 fatty acids is increasingly valued by consumers and can positively influence purchasing decisions, reflecting growing awareness of the health benefits associated with dietary ω-3 fatty acids [62].

Total ω-6 PUFA content decreased significantly with increasing BSFL inclusion, decreasing from 447.29 mg/100 DM in the control diet to 340.41 mg/100 DM at 9% inclusion (about 24% reduction). This decrease was associated with reductions in linoleic acid (18:2n-6) at higher inclusion levels and its downstream metabolites, particularly arachidonic acid (ARA; 20:4n-6), which decreased from 0.79 mg/100 at 0% to 0.26 mg/100 at 9% inclusion (approximately threefold reduction). As a result, the ω-3/ω-6 ratio increased significantly at 9% inclusion, rising from 0.14 in the control diet to approximately 0.17–0.19. Total PUFA content increased at 3% inclusion compared with the control but decreased at higher inclusion levels, reaching the lowest value at 9% inclusion. This reduction in total PUFA was primarily attributable to decreases in ω-6 PUFA, while eicosapentaenoic acid (EPA; 20:5n-3) increased with increasing BSFL inclusion.

In the context of human nutrition, this shift is particularly important, as modern Western diets are characterized by excessive ω-6 intake relative to ω-3 fatty acids, a dietary pattern associated with chronic low-grade inflammation and increased risk of cardiovascular and metabolic diseases [4,63]. Evidence suggests that the relative balance of PUFA, particularly the ω-3/ω-6 ratio, is a more important determinant of health outcomes than total PUFA intake alone [55,63]. Nutritional recommendations generally encourage increasing ω-3 fatty acid intake and reducing excessive ω-6 fatty acid consumption to achieve a more balanced dietary fatty acid profile, associated with reduced inflammation and improved cardiometabolic health [64,65]. Therefore, the improved ω-3/ω-6 ratio observed in the present study may represent a favourable characteristic for the development of nutritionally enhanced poultry meat products. Similar reductions in total PUFA alongside changes in fatty acid profiles have been reported in broiler meat from birds fed BSFL-based diets [39,45].

Although BSFL inclusion level was the primary driver of fatty acid modification in broiler breast meat, several differences among BSFL production sites were also evident. These production site-related effects likely reflect variation in larval substrate composition, as BSFL nutrient profiles are highly dependent on the organic waste on which larvae are reared [66]. In this study, BSFL-A and BSFL-B were reared on supermarket and childcare center waste, rich in mixed human food residues, whereas BSFL-C was mainly fed coffee ground waste, a substrate known for its low fat and high fiber content [67]. To our knowledge, this is the first study to assess how variation among BSFL production sites affects the fatty acid profile of broiler meat, providing new insights into substrate-driven differences in insect biomass. In the present study, capric acid (C10:0) differed significantly among BSFL production sites, with BSFL-A producing higher breast-meat concentrations than BSFL-C, whereas BSFL-B exhibited intermediate values. This difference may be related to variation in larval rearing substrates among BSFL production sites, as childcare and supermarket waste typically contain a broader range of cooked foods and animal-derived ingredients, which may contribute to increased deposition of medium-chain fatty acids in larvae. For MUFA, palmitoleic acid (16:1n-7), tended to be higher in BSFL-B compared with the other production site. The only significant PUFA effect across production sites was the ω-3/ω-6 ratio, which was lower in BSFL-C than in BSFL-A and BSFL-B. The inclusion of coffee grounds, which are low in ω-3 precursors, likely contributed to reduced enrichment of tissue ω-3 PUFA. As noted above, a balanced ω-3/ω-6 ratio is associated with reduced inflammation and improved cardiovascular outcomes in humans [4,63], and the higher ratios observed in BSFL-A and BSFL-B represent a nutritionally favorable profile. Although modest in magnitude, such differences underscore the importance of substrate standardization and characterization when BSFL are used as functional feed ingredients. Variability in BSFL derived from food-waste substrates may therefore reduce the reproducibility and consistency of broiler meat quality between production batches, representing an important consideration for industrial-scale application of food-waste–derived BSFL.

## 5. Conclusions

The present research showed that food-waste–derived full-fat BSFL can be included in broiler diets at levels up to 9% without adverse effects on carcass traits or physicochemical meat quality. The type of food waste used for larval rearing had minimal influence on most traits, although the tendencies for increased redness and ash content, particularly in BSFL-C, where coffee grounds comprised a major component of the substrate, suggest a potential substrate-related effect. Higher inclusion of BSFL increased breast meat protein content, whereas essential amino acid composition remained unchanged. In contrast, BSFL inclusion modified the fat nutritional profile of breast meat by selectively enriching short- and medium-chain saturated fatty acids, preserving the major long-chain saturated and monounsaturated fatty acids, and improving the balance between ω-3 and ω-6 polyunsaturated fatty acids through increased eicosapentaenoic acid (EPA) and reduced ω-6 PUFA. Production site also affected selected fatty acids and the ω-3/ω-6 ratio, indicating that larval substrate composition can influence broiler meat fatty acid profiles. These findings support BSFL as a sustainable feed ingredient for improving broiler meat nutritional quality, with further optimization possible through targeted substrate and processing strategies. Future studies should validate these findings across different genotypes and commercial conditions, with expanded sampling and sensory evaluation, and should further investigate the long-term nutritional, economic, and commercial implications of using food-waste–derived BSFL in poultry production systems.

## Figures and Tables

**Table 1:** Chemical and amino acid composition of full-fat black soldier fly larvae sourced from different production sites (BSFL-A, BSFL-B, and BSFL-C).

		BSFL	
Chemical compositions (g/kg DM)	BSFL-A	BSFL-B	BSFL-C
Dry matter	965	951.4	951.5
Ash	81.5	95.4	110
Crude Protein	261.7	263	263.6
Crude Fat	444.8	410	381.7
Neutral detergent fibre	314.8	326.7	294
Acid detergent fibre	94.8	88.2	91.2
Chitin	80.9	76.9	79.3
Essential Amino Acid, mg/gDM			
Arginine	16.0	14.5	15.0
Histidine	10.7	10.7	10.4
Isoleucine	13.6	12.2	12.7
Leucine	23.0	19.9	20.5
Lysine	18.4	17.2	18.0
Methionine	5.1	4.8	4.8
Phenylalanine	13.5	12.2	12.7
Threonine	15.0	12.9	13.5
Valine	20.0	19.0	19.8
Non- Essential Amino Acid, mg/g DM			
Alanine	23.4	21.9	21.5
Aspartic Acid	30.6	28.2	29.7
Cysteine	2.0	1.9	2.5
Glutamic Acid	30.8	36.1	30.6
Glycine	18.6	17.9	16.8
Proline	20.1	20.0	20.1
Serine	15.3	13.9	14.3
Tyrosine	21.4	19.5	19.2

BSFL-A = black soldier fly larvae sourced from production site A; BSFL-B = black soldier fly larvae sourced from production site B; BSFL-C = black soldier fly larvae sourced from production site C.

**Table 2:** Fatty acid composition (mg/100 g DM) of full-fat black soldier fly larvae sourced from different production sites (BSFL-A, BSFL-B, and BSFL-C).

Fatty Acid		BSFL	
Saturated Fatty Acids mg/100 g DM	BSFL-A	BSFL-B	BSFL-C
C8:0	29.7	20.5	9.6
C10:0	1062.0	899.8	587.1
C11:0	0.0	19.7	0.0
C12:0	10,286.3	12,538.3	9689.2
C13:0	0.0	18.5	0.0
C14:0	2126.5	2273.3	1968.9
C15:0	89.9	83.2	70.0
C16:0	6189.9	5606.1	6225.4
C17:0	107.2	62.3	67.2
C18:0	1614.9	1253.2	1402.3
C20:0	72.5	65.2	81.8
C23:0	38.9	37.8	38.0
ƩSFA	21,617.9	22,878.0	20,139.4
Monounsaturated Fatty Acids mg/100 g DM			
14:1n-5	125.8	112.9	73.4
C15:1	78.6	58.9	69.3
16:1n-7	1400.0	1206.3	1063.2
18:1n-9 cis	11,716.2	8030.6	8480.6
18:1n-7	431.0	259.4	284.1
18:1n-9 trans	66.9	0.0	0.0
20:1n-9	89.2	63.4	58.4
ƩMUFA	13,907.8	9731.6	10,029.1
Polyunsaturated Fatty Acids mg/100 g DM			
18:3n-3 ALA	941.8	837.6	896.4
18:4n-3	50.4	48.3	48.2
20:5n-3 EPA	183.0	163.8	94.3
18:2n-6 cis	5238.1	3743.1	5323.4
18:3n-6	0.0	0.0	16.5
20:2n-6	25.5	0.0	0.0
20:3n-6	81.7	70.6	66.9
22:6n-3 DHA	70.1	38.7	15.1
ω-3 PUFA	1245.4	1088.4	1054.1
ω-3 lcPUFA	253.1	202.5	109.4
ω-6 PUFA	5345.3	3813.7	5406.8
ω-3/ω-6	0.2	0.3	0.2
ƩPUFA	6565.2	4902.1	6460.8

BSFL-A = black soldier fly larvae sourced from production site A; BSFL-B = black soldier fly larvae sourced from production site B; BSFL-C = black soldier fly larvae sourced from production site C.

**Table 3:** Amino acid profile of experimental diets fed to the broiler chickens during the finisher phase (28–42 d).

Amino Acids				Experimental Diets						
Essential Amino Acid, mg/g DM	STD	A3%	A6%	A9%	B3%	B6%	B9%	C3%	C6%	C9%
Arginine	11.9	11.5	11.7	11.6	10.2	11.6	11.4	12.7	13.2	12.9
Histidine	5.7	5.9	5.8	5.5	5.1	5.9	6.0	8.3	5.7	6.2
Isoleucine	7.0	6.9	7.3	7.1	7.2	6.9	7.4	6.5	7.7	7.0
Leucine	12.6	12.1	12.3	11.9	12.8	11.3	11.7	11.9	12.6	12.1
Lysine	9.8	10.0	10.2	10.3	8.0	9.2	8.7	10.3	10.9	10.3
Methionine	4.8	5.3	5.3	5.1	5.3	5.0	5.8	5.5	5.8	5.3
Phenylalanine	7.7	7.9	8.0	7.9	8.1	7.7	7.3	7.3	8.4	7.9
Threonine	8.8	9.0	9.0	8.8	8.6	8.7	9.6	8.9	9.7	8.8
Valine	8.7	9.3	9.2	8.8	9.0	8.6	9.1	8.9	9.5	8.9
Non- Essential Amino Acid, mg/g DM										
Alanine	8.3	8.6	8.5	9.0	9.0	8.4	9.1	8.6	9.3	8.9
Aspartic Acid	14.8	14.9	14.9	14.3	14.1	14.0	13.7	14.3	15.9	15.1
Cysteine	2.0	2.2	2.0	2.2	2.2	2.0	1.9	2.1	2.2	2.1
Glutamic Acid	25.4	24.6	24.4	25.1	24.8	23.9	21.9	23.3	25.8	24.2
Glycine	9.8	10.0	8.8	8.7	9.8	8.8	8.7	9.8	9.3	8.3
Proline	11.7	12.2	12.2	12.2	12.3	11.6	11.7	11.7	12.5	11.6
Serine	8.5	8.7	8.5	8.7	8.7	8.4	8.1	8.4	8.9	8.5
Tyrosine	4.6	4.7	5.2	5.2	5.3	4.9	5.4	4.8	5.5	5.4

STD = standard finisher diet (0% black soldier fly larvae). A3%, A6%, and A9% = BSFL sourced from production site A included at 3%, 6%, and 9% of the diet, respectively; B3%, B6%, and B9% = site B at 3%, 6%, and 9%; C3%, C6%, and C9% = site C at 3%, 6%, and 9%.

**Table 4:** Fatty acid profile of experimental diets fed to the broiler chickens during the finisher phase (28–42 d).

Fatty Acid		Experimental Diets								
Saturated Fatty Acids	STD	A3%	A6%	A9%	B3%	B6%	B9%	C3%	C6%	C9%
C8:0	0.0	1.2	1.9	3.2	1.5	1.8	2.8	0.0	0.0	0.0
C10:0	1.3	28.7	67.8	136.6	30.4	62.0	100.6	18.1	36.7	53.2
C12:0	8.4	285.6	692.6	1380.0	411.0	836.2	1164.5	304.6	651.4	950.0
C14:0	36.1	84.2	162.8	302.7	110.8	182.3	260.5	90.1	153.9	201.2
C15:0	8.2	8.4	10.7	17.6	11.5	11.1	12.9	8.4	9.3	9.9
C16:0	828.8	799.4	1015.3	1473.6	1001.0	1057.5	1142.6	908.1	1061.0	1058.8
C17:0	16.5	15.5	17.9	24.3	17.5	16.2	15.2	15.6	14.9	11.7
C18:0	354.1	307.8	334.5	412.1	365.2	335.7	313.9	334.7	320.3	257.6
C20:0	26.4	21.5	22.8	27.9	26.7	23.9	22.9	24.6	24.9	21.4
C23:0	9.7	8.7	9.2	15.2	10.4	10.2	9.3	8.5	9.5	8.2
C24:0	15.9	12.0	11.4	13.2	14.8	12.2	10.2	12.7	12.5	9.6
Ʃ SFA	1305.5	1573.0	2346.9	3806.4	2000.7	2549.2	3055.5	1725.5	2294.5	2581.8
Monounsaturated Fatty Acids										
14:1n-5	3.7	6.4	10.6	17.8	7.5	10.4	14.4	5.5	6.9	7.5
C15:1	11.8	11.6	13.6	24.7	14.3	14.1	14.7	12.1	13.1	13.7
16:1n-7	22.2	55.0	106.4	199.0	63.7	105.1	155.2	51.7	85.6	109.3
18:1n-9 cis	1561.8	1483.2	1892.6	2687.0	1733.0	1802.4	1866.4	1603.2	1715.5	1618.4
18:1n-7	87.3	79.9	96.8	125.5	94.4	94.4	96.3	84.8	90.0	77.8
20:1n-9	17.3	16.2	20.1	27.4	18.1	20.4	21.8	16.9	17.9	16.8
22:1n-9	9.0	7.4	6.3	5.8	10.1	6.0	4.1	6.8	8.6	5.2
Ʃ MUFA	1726.3	1671.3	2154.6	3087.3	1941.1	2052.7	2172.9	1781.0	1937.7	1848.6
Polyunsaturated Fatty Acids										
18:3n-3 ALA	266.2	224.4	246.7	302.2	261.8	257.1	246.3	250.9	247.6	223.1
18:4n-3	13.3	14.0	15.4	25.6	16.3	16.1	15.5	13.2	15.3	14.7
20:5n-3 EPA	0.0	7.1	13.6	25.4	3.1	13.2	16.8	2.2	8.9	10.6
18:2n-6 cis	2597.9	2098.8	2269.0	2801.9	2498.7	2306.8	2066.3	2406.2	2341.0	2075.3
20:3n-6	0.0	3.5	6.3	11.7	3.3	6.8	8.7	3.7	5.0	6.5
ω-3 PUFA	279.4	245.5	275.6	353.3	281.1	286.4	284.7	264.1	271.7	248.4
ω-3 lcPUFA	0.0	7.1	13.6	25.4	3.1	13.2	22.9	3.8	8.9	10.6
ω-6 PUFA	2607.6	2107.9	2279.4	2813.5	2500.5	2313.6	2079.8	2418.9	2348.6	2083.4
ω-3/ω-6	0.1	0.1	0.1	0.1	0.1	0.1	0.1	0.1	0.1	0.1
Ʃ PUFA	2883.6	2350.1	2551.0	3166.8	2779.8	2600.0	2359.7	2679.6	2620.3	2330.1

STD = standard finisher diet (0% black soldier fly larvae). A3%, A6%, and A9% = BSFL sourced from production site A included at 3%, 6%, and 9% of the diet, respectively; B3%, B6%, and B9% = site B at 3%, 6%, and 9%; C3%, C6%, and C9% = site C at 3%, 6%, and 9%; ΣSFA = total saturated fatty acids; ΣMUFA = total monounsaturated fatty acids; ω-3 PUFA = total omega-3 polyun-saturated fatty acids; ω-3 lcPUFA = long-chain omega-3 polyunsaturated fatty acids; ω-6 PUFA = total omega-6 polyun-saturated fatty acids; ω-3/ω-6 = ratio of omega-3 to omega-6 PUFA; ΣPUFA = total polyunsaturated fatty acids.

**Table 5:** Effects of BSFL inclusion rate and production site on breast meat quality parameters of broiler chickens.

Interactions		Breast Meat Quality Parameters											
BSFL	Inclusion Rate %	LW (g)	CCW (g)	DP (%)	pH	WHC %	SF N	CL %	L*	a*	b*	Hue (°)	Chroma
BSFL-A	0	3338	2429	72.8	5.85	73.5	23.0	25.1	47.8	2.7	3.2	39.7	4.19
	3	3339	2459	73.7	5.85	73.0	22.6	25.5	47.8	2.6	3.0	41.9	3.98
	6	3188	2335	73.3	5.92	72.5	22.7	25.0	47.4	2.6	2.8	42.8	3.89
	9	3213	2253	70.7	5.88	72.6	23.3	26.0	46.5	2.4	3.1	38.8	3.94
BSFL-B	0	3234	2376	73.5	5.90	73.0	22.9	25.7	48.5	2.7	3.5	38.5	4.47
	3	3249	2393	73.7	5.81	72.9	20.8	26.5	47.7	2.7	3.3	40.2	4.28
	6	3342	2440	73.0	5.84	73.6	22.6	25.4	48.8	2.6	3.1	41.5	4.06
	9	3211	2339	72.9	6.01	72.2	22.9	26.7	41.1	2.7	2.8	45.3	4.00
BSFL-C	0	3258	2373	72.8	5.87	73.0	22.6	26.4	47.9	3.6	3.7	43.4	5.21
	3	3281	2407	73.4	5.92	72.9	21.8	27.2	47.6	2.8	2.8	46.5	4.08
	6	3284	2431	74.0	5.87	71.9	22.8	26.0	46.5	3.3	3.4	41.4	4.81
	9	3290	2449	74.5	5.85	73.1	23.8	27.6	47.1	2.9	3.1	42.4	4.22
SEM		85.11	59.55	1.076	0.057	0.692	1.513	1.041	2.246	0.378	0.405	4.030	0.470
*p*-value		0.738	0.304	0.542	0.267	0.669	0.996	1.000	0.598	0.931	0.879	0.853	0.921
Main effect													
BSFL	BSFL-A	3270	2369	72.6	5.87	72.9	22.9	25.4	47.4	2.56	3.00	40.8	4.00
	BSFL-B	3259	2387	73.3	5.89	72.9	22.3	26.1	46.5	2.69	3.17	41.4	4.20
	BSFL-C	3278	2415	73.7	5.88	72.7	22.8	26.8	47.3	3.13	3.25	43.4	4.58
SEM		42.56	29.78	0.538	0.029	0.346	0.756	0.521	1.123	0.189	0.202	2.025	0.235
*p*-value		0.950	0.556	0.367	0.917	0.879	0.856	0.174	0.847	0.089	0.682	0.630	0.215
Main effect													
Inclusion rate %	0	3277	2393	73.02	5.87	73.2	22.8	25.8	48.0	3.00	3.47	40.6	4.62
	3	3290	2420	73.58	5.86	72.9	21.8	26.4	47.7	2.70	3.01	42.9	4.12
	6	3271	2402	73.45	5.88	72.7	22.7	25.5	47.6	2.82	3.10	41.9	4.25
	9	3238	2347	72.67	5.91	72.6	23.3	26.8	44.9	2.65	2.98	42.2	4.05
SEM		49.14	34.38	0.622	0.033	0.399	0.874	0.601	1.297	0.218	0.234	2.327	0.271
*p*-value		0.895	0.492	0.719	0.747	0.763	0.643	0.420	0.301	0.667	0.446	0.925	0.453

BSFL-A = black soldier fly larvae sourced from production site A; BSFL-B = black soldier fly larvae sourced from production site B; BSFL-C = black soldier fly larvae sourced from production site C; LW = Live weight; CCW = Cold carcass weight; DP = Dressing percentage; WHC = Water holding capacity; SF = Shear force; CL = Cooking loss; L* = Lightness; a* = Redness; b* = Yellowness; SEM = Standard Error of Mean.

**Table 6:** Effects of BSFL inclusion rate and production site on meat proximate analyses of broiler chicken.

Interactions		Meat Approximate Analyses			
BSFL	Inclusion Rate	Moisture%	Protein% (As Is)	Fat% (As Is)	Ash% (As Is)
BSFL-A	0	76.3	19.5	1.90	1.06
	3	75.3	21.2	1.05	1.28
	6	75.4	20.9	1.47	1.27
	9	75.3	21.2	1.62	1.29
BSFL-B	0	75.3	19.2	1.43	1.31
	3	75.1	21.4	1.82	1.20
	6	75.5	20.8	1.24	1.23
	9	74.8	22.2	1.15	1.19
BSFL-C	0	75.4	21.3	1.43	1.42
	3	75.3	21.3	1.46	1.33
	6	74.6	21.4	1.78	1.33
	9	75.0	21.7	1.49	1.28
SEM		0.345	0.662	0.303	0.080
*p*-value		0.407	0.551	0.302	0.237
Main effect					
BSFL	BSFL-A	75.6	20.7	1.50	1.22
	BSFL-B	75.2	20.9	1.41	1.23
	BSFL-C	75.1	21.4	1.54	1.34
SEM		0.172	0.331	0.152	0.040
*p*-value		0.137	0.301	0.820	0.084
Main effect					
Inclusion rate	0	75.6	20.0 ^b^	1.59	1.26
	3	75.2	21.3 ^ab^	1.44	1.27
	6	75.2	21.1 ^ab^	1.48	1.28
	9	75.0	21.7 ^a^	1.42	1.25
SEM		0.120	0.382	0.175	0.047
*p*-value		0.174	0.017	0.912	0.982

BSFL-A = black soldier fly larvae sourced from production site A; BSFL-B = black soldier fly larvae sourced from production site B; BSFL-C = black soldier fly larvae sourced from production site C; SEM = Standard Error of Mean; ^a,b^ Values with different superscripts differ (*p* ≤ 0.05).

**Table 7:** Effect of the dietary inclusion of BSFL on essential amino acid composition (mg/g dry matter) of broiler breast meat at 42 days of age.

Interactions		Essential Amino Acid, mg/g DM								
BSFL	Inclusion Rate	Arginine	Histidine	Isoleucine	Leucine	Lysine	Methionine	Phenylalanine	Threonine	Valine
BSFL-A	0	57.8	28.5	32.0	54.4	77.9	17.8	29.2	33.8	36.6
	3	50.4	24.2	30.7	54.1	75.2	17.8	28.9	34.3	35.8
	6	60.5	30.0	32.7	54.0	80.2	18.7	29.6	34.0	36.4
	9	50.2	24.3	33.5	55.0	79.9	18.8	29.6	34.9	36.8
BSFL-B	0	57.8	28.5	32.0	54.4	77.9	17.8	29.2	33.8	36.6
	3	51.5	27.6	31.2	52.3	73.1	16.5	27.7	32.9	35.0
	6	53.2	27.2	31.0	52.5	74.0	17.1	28.1	33.1	35.2
	9	51.9	24.9	31.9	53.8	78.7	19.6	29.3	34.0	36.4
BSFL-C	0	57.8	28.5	32.0	54.4	77.9	17.8	29.2	33.8	36.6
	3	53.2	27.1	31.1	53.1	76.3	18.7	28.6	34.0	35.8
	6	52.2	24.9	31.9	54.5	76.0	17.5	29.0	33.9	36.5
	9	56.5	27.6	33.2	54.1	78.7	18.0	29.5	34.1	36.3
SEM		4.367	2.104	1.113	1.091	3.245	0.777	0.689	0.673	0.899
*p*-value		0.573	0.249	0.916	0.889	0.909	0.121	0.893	0.907	0.955
Main effects										
BSFL	BSFL-A	54.7	26.8	32.2	54.4	78.3	18.3	29.3	34.2	36.4
	BSFL-B	53.6	27.0	31.6	53.2	75.9	17.8	28.6	33.4	35.8
	BSFL-C	54.9	27.0	32.0	54.0	77.2	18.0	29.1	33.9	36.3
SEM		1.891	0.911	0.482	0.472	1.405	0.337	0.298	0.292	0.389
*p*-value		0.865	0.972	0.592	0.233	0.489	0.534	0.202	0.157	0.491
Main effects										
Inclusion rate	0	57.8	28.5	32.0	54.4	77.9	17.8	29.2	33.8	36.6
	3	51.7	26.3	31.0	53.1	74.9	17.7	28.4	33.7	35.5
	6	55.3	27.4	31.9	53.6	76.7	17.8	28.9	33.7	36.0
	9	52.9	25.6	32.9	54.3	79.1	18.8	29.5	34.3	36.5
SEM		2.377	1.145	0.606	0.594	1.766	0.423	0.375	0.367	0.489
*p*-value		0.206	0.199	0.160	0.348	0.328	0.135	0.204	0.437	0.305

BSFL-A = black soldier fly larvae sourced from production site A; BSFL-B = black soldier fly larvae sourced from production site B; BSFL-C = black soldier fly larvae sourced from production site C; SEM = Standard Error of Mean.

**Table 8:** Effect of the dietary inclusion of BSFL on non- essential amino acid composition (mg/g dry matter) of broiler breast meat at 42 days of age.

Interactions		Non- Essential Amino Acid, mg/g DM							
BSFL	Inclusion Rate	Alanine	Aspartic Acid	Cysteine	Glutamic Acid	Glycine	Proline	Serine	Tyrosine
BSFL-A	0	36.3	64.6	6.3	82.0	31.6	26.7	29.0	25.4
	3	36.4	64.6	6.2	81.9	32.6	26.9	29.7	25.2
	6	36.7	63.8	6.1	83.1	30.8	26.8	28.6	25.7
	9	36.9	66.1	6.4	85.9	31.3	27.4	29.2	25.7
BSFL-B	0	36.3	64.6	6.3	82.0	31.6	26.7	29.0	25.4
	3	35.3	62.2	5.9	78.5	30.3	25.3	27.7	24.5
	6	35.4	62.6	5.6	80.8	30.5	25.9	27.9	24.4
	9	35.8	64.1	5.7	81.5	30.5	26.6	28.3	25.4
BSFL-C	0	36.3	64.6	6.3	82.0	31.6	26.7	29.0	25.4
	3	36.7	64.0	6.2	81.7	33.3	26.7	30.9	25.8
	6	36.5	64.7	6.2	83.9	31.2	26.3	28.7	25.8
	9	36.3	64.7	6.1	86.0	30.6	26.8	29.4	25.6
SEM		0.776	1.308	0.255	2.439	0.643	0.470	0.949	0.562
*p*-value		0.908	0.869	0.709	0.934	0.135	0.482	0.667	0.645
Main effects									
BSFL	BSFL-A	36.5	64.8	6.2	83.2	31.6 ^ab^	27.0 ^a^	29.1	25.5
	BSFL-B	35.7	63.4	5.9	80.7	30.7 ^b^	26.1 ^b^	28.2	24.9
	BSFL-C	36.5	64.5	6.2	83.4	31.7 ^a^	26.6 ^ab^	29.5	25.7
SEM		0.336	0.566	0.110	1.056	0.278	0.204	0.411	0.243
*p*-value		0.151	0.192	0.051	0.137	0.034	0.017	0.086	0.073
Main effects									
Inclusion rate	0	36.3	64.6	6.3	82.0	31.6 ^ab^	26.7	29.0	25.4
	3	36.1	63.6	6.1	80.7	32.1 ^a^	26.3	29.5	25.2
	6	36.2	63.7	5.9	82.6	30.8 ^b^	26.3	28.4	25.3
	9	36.4	65.0	6.1	84.5	30.8 ^b^	26.9	29.0	25.6
SEM		0.422	0.712	0.139	1.327	0.350	0.256	0.516	0.306
*p*-value		0.978	0.359	0.277	0.182	0.021	0.176	0.458	0.806

BSFL-A = black soldier fly larvae sourced from production site A; BSFL-B = black soldier fly larvae sourced from production site B; BSFL-C = black soldier fly larvae sourced from production site C; SEM = Standard Error of Mean; ^a,b^ Individual amino acid values with different superscripts differ (*p* ≤ 0.05).

**Table 9:** Effect of the dietary inclusion of BSFL on the saturated fatty acid composition (mg/g dry matter) of broiler breast meat at 42 days of age.

Interactions		Saturated Fatty Acids										
BSFL	Inclusion Rate	C10:0	C12:0	C14:0	C15:0	C16:0	C17:0	C18:0	C19:0	C20:0	C23:0	ƩSFA
BSFL-A	0	0.00	1.47 ^d^	9.93	2.44	328.29	3.31	145.87	2.49	2.04	2.22	498.06
	3	1.33	30.58 ^c^	21.23	2.61	359.02	4.00	153.81	2.46	2.40	2.63	580.07
	6	1.92	45.48 ^b^	24.43	2.28	313.60	3.13	128.51	2.36	1.77	2.27	525.74
	9	3.20	77.73 ^a^	35.60	2.69	359.40	3.43	137.64	2.47	1.85	1.94	625.94
BSFL-B	0	0.00	1.47 ^d^	9.93	2.44	328.29	3.31	145.87	2.49	2.04	2.22	498.06
	3	1.45	42.96 ^c^	28.64	3.19	490.18	4.69	196.64	3.99	2.87	2.82	777.42
	6	2.26	62.08 ^b^	32.03	2.66	416.39	3.21	149.46	3.03	2.23	2.49	675.84
	9	2.50	65.24 ^a^	31.27	2.31	315.30	2.68	120.93	1.55	1.66	2.23	545.66
BSFL-C	0	0.00	1.47 ^d^	9.93	2.44	328.29	3.31	145.87	2.49	2.04	2.22	498.06
	3	0.64	23.08 ^c^	16.99	2.18	312.78	3.23	136.84	2.50	1.99	2.16	502.38
	6	1.49	53.84 ^b^	29.32	2.59	382.43	3.48	157.90	3.10	2.26	2.28	638.68
	9	2.46	93.51 ^a^	41.16	2.82	408.10	3.29	154.48	3.19	2.33	2.49	713.83
SEM		0.270	6.870	3.336	0.344	44.205	0.498	16.890	0.693	0.270	0.197	71.158
*p*-value		0.329	0.034	0.079	0.459	0.098	0.470	0.180	0.435	0.163	0.212	0.096
Main effects												
BSFL	BSFL-A	1.61 ^a^	38.81	22.80	2.50	340.08	3.47	141.46	2.44	2.01	2.27	557.45
	BSFL-B	1.55 ^ab^	42.94	25.47	2.65	387.54	3.47	153.23	2.77	2.20	2.44	624.25
	BSFL-C	1.15 ^b^	42.97	24.35	2.51	357.90	3.33	148.77	2.82	2.15	2.29	588.24
SEM		0.135	3.435	1.668	0.172	22.102	0.249	8.445	0.347	0.135	0.098	35.579
*p*-value		0.036	0.618	0.528	0.789	0.315	0.898	0.612	0.710	0.604	0.394	0.419
Main effects												
Inclusion rate	0	0.00 ^d^	1.47 ^d^	9.93 ^c^	2.44	328.29	3.31	145.87	2.49	2.04	2.22	498.06
	3	1.14 ^c^	32.21 ^c^	22.29 ^b^	2.66	387.32	3.97	162.43	2.98	2.42	2.54	619.96
	6	1.89 ^b^	53.80 ^b^	28.59 ^b^	2.51	370.81	3.27	145.29	2.83	2.09	2.35	613.42
	9	2.72 ^a^	78.82 ^a^	36.01 ^a^	2.61	360.93	3.13	137.68	2.40	1.95	2.22	628.48
SEM		0.156	3.966	1.926	0.198	25.522	0.288	9.751	0.400	0.156	0.114	41.083
*p*-value		<0.0001	<0.0001	<0.0001	0.862	0.422	0.176	0.339	0.702	0.171	0.176	0.092

BSFL-A = black soldier fly larvae sourced from production site A; BSFL-B = black soldier fly larvae sourced from production site B; BSFL-C = black soldier fly larvae sourced from production site C; SEM = Standard Error of Mean; ^a–d^ Individual fatty acid values with different superscripts differ (*p* ≤ 0.05); ΣSFA = total saturated fatty acids.

**Table 10:** Effect of the dietary inclusion of BSFL on the monounsaturated fatty acid composition (mg/g dry matter) of broiler breast meat at 42 days of age.

Interactions		Monounsaturated Fatty Acids.								
BSFL	Inclusion Rate	14:1n-5	C15:1	16:1n-7	18:1n-9 cis	18:1n-7	20:1n-9	22:1n-9	24:1n-9	ƩMUFA
BSFL-A	0	1.67	4.59	43.62	482.74	34.66	5.25	0.65	0.95	574.14
	3	3.10	5.53	51.74	543.66	38.74	5.83	0.24	1.06	649.90
	6	3.48	4.61	50.19	476.12	34.71	5.22	0.16	0.87	575.35
	9	5.18	4.44	67.36	554.17	40.09	5.64	0.33	0.76	677.98
BSFL-B	0	1.67	4.59	43.62	482.74	34.66	5.25	0.65	0.95	574.14
	3	4.48	5.43	80.86	754.31	51.90	7.84	0.19	1.02	906.03
	6	5.43	5.34	83.23	612.52	47.36	6.77	0.40	1.50	762.54
	9	4.55	4.75	59.60	474.75	39.14	5.56	0.47	1.39	590.21
BSFL-C	0	1.67	4.59	43.62	482.74	34.66	5.25	0.65	0.95	574.14
	3	2.37	4.67	42.49	467.79	34.11	5.08	0.70	1.06	558.27
	6	3.87	5.00	58.55	549.53	41.03	6.23	0.50	1.24	665.94
	9	5.60	5.00	70.80	606.13	43.88	6.92	0.44	1.49	740.26
SEM		0.564	0.291	8.725	72.066	4.320	0.695	0.239	0.465	85.502
*p*-value		0.081	0.216	0.059	0.144	0.167	0.136	0.898	0.968	0.130
Main effects										
BSFL	BSFL-A	3.36	4.80	53.23	514.17	37.05	5.49	0.34	0.91	619.34
	BSFL-B	4.03	5.03	66.83	581.08	43.26	6.35	0.43	1.22	708.23
	BSFL-C	3.38	4.82	53.86	526.55	38.42	5.87	0.57	1.18	634.65
SEM		0.282	0.146	4.363	36.033	2.160	0.348	0.120	0.232	42.75
*p*-value		0.166	0.464	0.053	0.383	0.111	0.219	0.395	0.593	0.298
Main effects										
Inclusion rate	0	1.67 ^c^	4.59	43.62 ^b^	482.74	34.66	5.25	0.65	0.95	574.14
	3	3.32 ^b^	5.21	58.36 ^ab^	588.59	41.58	6.25	0.38	1.04	704.73
	6	4.26 ^ab^	4.98	63.99 ^a^	546.06	41.03	6.07	0.35	1.20	667.94
	9	5.11 ^a^	4.73	65.92 ^a^	545.02	41.04	6.04	0.41	1.21	669.48
SEM		0.325	0.168	5.038	41.607	2.494	0.401	0.138	0.268	49.36
*p*-value		<0.0001	0.056	0.011	0.357	0.168	0.310	0.421	0.878	0.288

BSFL-A = black soldier fly larvae sourced from production site A; BSFL-B = black soldier fly larvae sourced from production site B; BSFL-C = black soldier fly larvae sourced from production site C; SEM = Standard Error of Mean; ^a,b,c^ Values with different superscripts differ (*p* ≤ 0.05); ΣMUFA = total monounsaturated fatty acids.

**Table 11:** Effect of the dietary inclusion of BSFL on the polyunsaturated fatty acid composition (mg/g dry matter) of broiler breast meat at 42 days of age.

Interactions		Polyunsaturated Fatty Acids														
BSFL	Inclusion Rate	16:3n3	18:3n-3 ALA	18:4n-3	20:5n-3 EPA	22:5n-3 DPA	18:2n-6 cis	20:2n-6	20:3n-6	20:4n-6 ARA	22:4n-6	ω-3 PUFA	ω-3 lcPUFA	ω-6 PUFA	ω-3/ω-6	ƩPUFA
BSFL-A	0	8.04	32.31	4.21	2.64	13.51	371.47	10.08	60.90	0.79	4.05	60.96	24.45	447.29	0.14 ^f^	506.21
	3	8.01	35.75	4.19	3.76	14.98	389.50	9.88	55.63	0.32	3.28	66.93	26.99	458.61	0.15 ^cd^	523.67
	6	7.37	25.09	3.81	3.74	14.71	288.39	8.81	50.32	0.40	2.92	56.88	27.99	350.84	0.16 ^b^	406.27
	9	7.72	24.15	3.66	4.95	15.53	275.06	8.35	48.89	0.12	2.41	59.39	31.58	334.84	0.18 ^a^	393.60
BSFL-B	0	8.04	32.31	4.21	2.64	13.51	371.47	10.08	60.90	0.79	4.05	60.96	24.45	447.29	0.14 ^f^	506.21
	3	9.11	45.83	4.39	3.65	16.29	486.11	11.46	63.14	0.10	3.63	79.96	29.74	564.44	0.14 ^ef^	642.05
	6	8.20	30.25	3.61	4.85	15.81	330.89	9.77	52.89	0.18	2.69	63.78	29.92	396.41	0.16 ^b^	458.62
	9	6.93	20.41	4.13	5.10	14.28	226.10	8.37	44.16	0.36	1.91	53.61	29.07	280.90	0.19 ^a^	333.06
BSFL-C	0	8.04	32.31	4.21	2.64	13.51	371.47	10.08	60.90	0.79	4.05	60.96	24.45	447.29	0.14 ^f^	506.21
	3	8.37	29.73	3.89	3.09	14.28	340.26	9.88	56.39	0.68	3.60	59.11	25.48	410.83	0.15 ^de^	468.42
	6	9.07	31.76	4.07	3.97	15.08	358.08	10.87	59.06	0.68	3.51	62.94	27.12	432.20	0.15 ^de^	493.35
	9	8.89	31.04	3.70	4.39	14.93	338.43	10.58	53.15	0.29	3.03	62.70	27.96	405.48	0.16 ^bc^	466.49
SEM		0.677	4.850	0.250	0.361	1.290	48.045	0.924	4.916	0.230	0.402	6.835	2.518	52.770	0.004	59.135
*p*-value		0.4498	0.229	0.518	0.556	0.938	0.2539	0.483	0.708	0.735	0.749	0.476	0.936	0.269	0.002	0.286
Main effects																
BSFL	BSFL-A	7.78	29.32	3.97	3.77	14.68	331.11	9.28	53.93	0.41	3.16	61.04	27.75	397.89	0.16 ^a^	457.44
	BSFL-B	8.07	32.20	4.08	4.06	14.97	353.64	9.92	55.27	0.36	3.07	64.58	28.29	422.26	0.16 ^a^	484.99
	BSFL-C	8.60	31.21	3.96	3.52	14.45	352.06	10.36	57.38	0.61	3.55	61.43	26.26	423.95	0.15 ^b^	483.62
SEM		0.339	2.425	0.125	0.180	0.645	24.023	0.462	2.458	0.115	0.201	3.417	1.259	26.385	0.002	29.567
*p*-value		0.2363	0.697	0.748	0.119	0.847	0.7611	0.263	0.610	0.266	0.211	0.726	0.499	0.738	<0.0001	0.760
Main effects																
Inclusion rate	0	8.04	32.31 ^ab^	4.21	2.64 ^c^	13.51	371.47 ^ab^	10.08	60.90 ^a^	0.79 ^a^	4.05 ^a^	60.96	24.45	447.29 ^ab^	0.14 ^d^	506.21 ^ab^
	3	8.50	37.10 ^a^	4.16	3.50 ^b^	15.18	405.29 ^a^	10.41	58.39 ^ab^	0.37 ^ab^	3.5 ^ab^	68.66	27.40	477.96 ^a^	0.15 ^c^	544.71 ^a^
	6	8.21	29.03 ^ab^	3.83	4.18 ^ab^	15.20	325.78 ^ab^	9.82	54.09 ^ab^	0.42 ^ab^	3.04 ^bc^	61.20	28.34	393.15 ^ab^	0.16 ^b^	452.75 ^ab^
	9	7.85	25.20 ^b^	3.83	4.81 ^a^	14.91	279.89 ^b^	9.10	48.73 ^b^	0.26 ^b^	2.45 ^c^	58.56	29.54	340.41 ^b^	0.17 ^a^	397.72 ^b^
SEM		0.391	2.800	0.144	0.208	0.745	27.739	0.534	2.838	0.133	0.232	3.946	1.454	30.467	0.002	34.142
*p*-value		0.6880	0.028	0.121	<0.0001	0.330	0.0134	0.363	0.020	0.038	<0.0001	0.307	0.094	0.012	<0.0001	0.020

BSFL-A = black soldier fly larvae sourced from production site A; BSFL-B = black soldier fly larvae sourced from production site B; BSFL-C = black soldier fly larvae sourced from production site C; SEM = standard error of the mean; ^a–f^ Values with different superscripts differ (*p* ≤ 0.05); ω-3 PUFA = total omega-3 polyunsaturated fatty acids; ω-3 lcPUFA = long-chain omega-3 polyunsaturated fatty acids; ω-6 PUFA = total omega-6 polyunsaturated fatty acids; ω-3/ω-6 ratio = ratio of omega-3 to omega-6 PUFA; ΣPUFA = total polyunsaturated fatty acids.

## Data Availability

The original contributions presented in this study are included in the article. Further inquiries can be directed to the corresponding author.
